# Angioma Serpiginosum in a Young Woman Following COVID-19 Infection

**DOI:** 10.7759/cureus.113824

**Published:** 2026-08-02

**Authors:** Samantha R Steiss, Sama Alazawi, Scott A Norton

**Affiliations:** 1 Transitional Year Program, Tripler Army Medical Center, Honolulu, USA; 2 Department of Dermatology, Walter Reed National Military Medical Center, Bethesda, USA; 3 Department of Dermatology, Uniformed Services University of the Health Sciences, Bethesda, USA

**Keywords:** acquired vasculopathy, angioma serpiginosum, case report, clinical dermatology, covid-19, cutaneous vasculopathy, sars-cov-2 infection

## Abstract

Angioma serpiginosum (AS) is an uncommon, benign capillary malformation that typically presents in childhood as nonblanching, red-purple serpiginous macules, usually on a single extremity. We report the case of a 25-year-old woman who experienced the abrupt onset of numerous bilateral red-violaceous serpiginous and reticular patches on the extensor surfaces of all extremities one month following COVID-19 infection. Follow-up over three years showed no change in distribution or morphology. Atypical features of her presentation include onset in young adulthood and a bilaterally symmetric distribution. The close temporal association with SARS-CoV-2 infection raises the possibility that her AS was a post-COVID-19-acquired vasculopathy, thereby expanding the spectrum of COVID-19-associated vascular sequelae.

## Introduction

COVID-19 is a viral infection caused by SARS-CoV-2 that produces a wide range of systemic effects, with manifestations extending to the skin as well as other organs [1]. Cutaneous manifestations typically fall into one of six phenotypes (urticarial rash, confluent erythematous/maculopapular/morbilliform rash, papulovesicular rash, chilblain-like acral pattern, livedo reticularis/racemosa-like pattern, or a purpuric vasculitic pattern) [1]. Although vascular patterns represent the least common cutaneous manifestations of COVID-19, the close temporal relationship between COVID-19 infection and the emergence of several unusual acquired vascular disorders suggests a plausible causal role for the virus in triggering sudden-onset, rare vasculopathies [1-3]. This report describes a unique case of angioma serpiginosum (AS) associated with COVID-19.

## Case presentation

**Figure 1:**
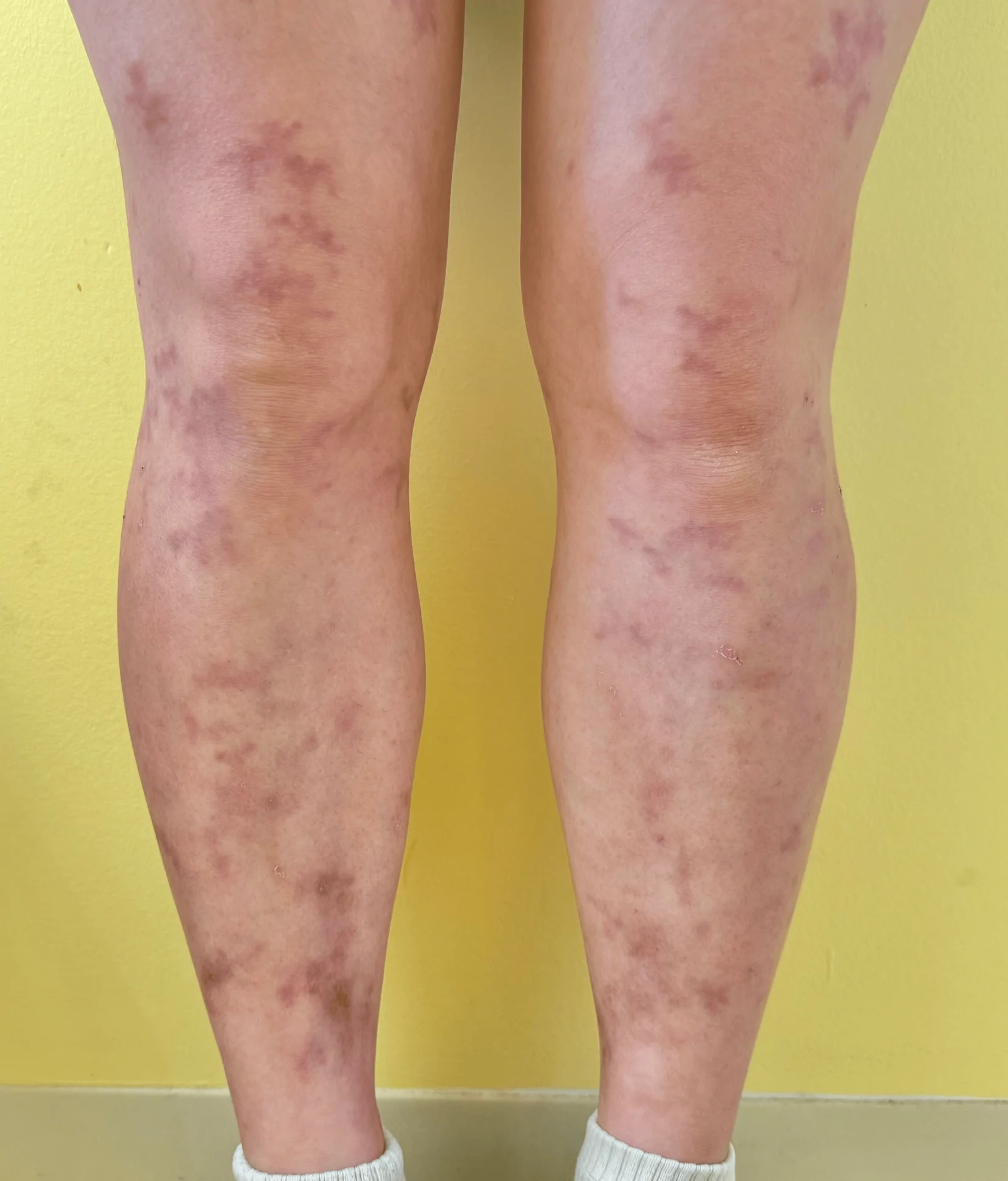
Red-violaceous reticular patches in a serpiginous pattern on the patient’s bilateral anterior lower extremities.

**Figure 2:**
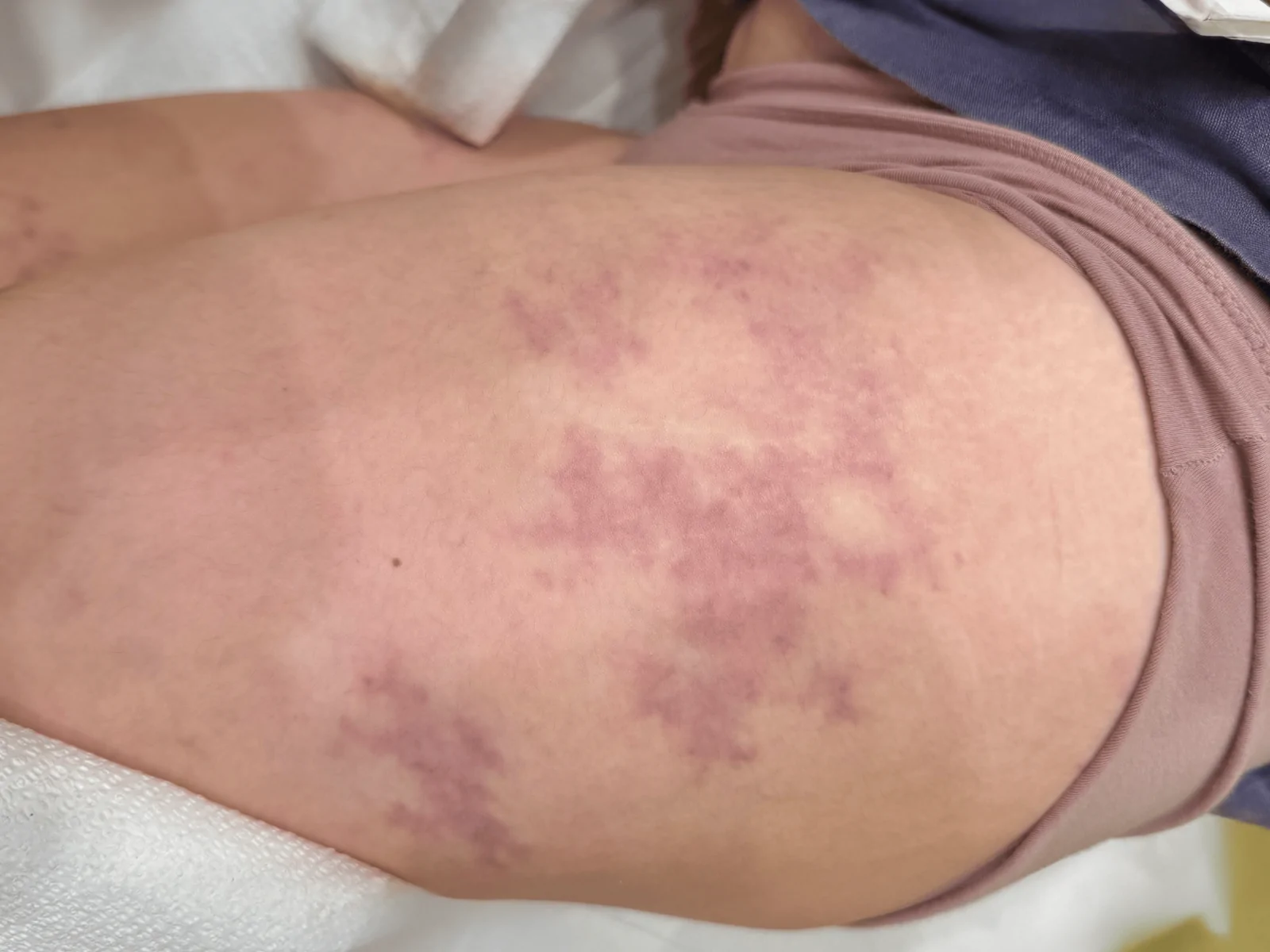
Fixed violaceous patches with stellate borders on the patient’s left lateral thigh.

We report an otherwise healthy 25-year-old woman with a three-year history of fixed, irregular red patches on the anterolateral surfaces of her arms and legs. All lesions arose abruptly approximately one month after her first episode of COVID-19. Since then, the lesions have remained unchanged in size, distribution, and number. She has approximately 40 red-violaceous serpiginous and reticular patches with irregular, stellate borders on the anterolateral surfaces of all extremities, most prominently on the extensor surfaces of the knees and elbows (Figure 1, Figure 2). The lesions are asymptomatic, nontender, and spare flexural surfaces.

**Figure 3:**
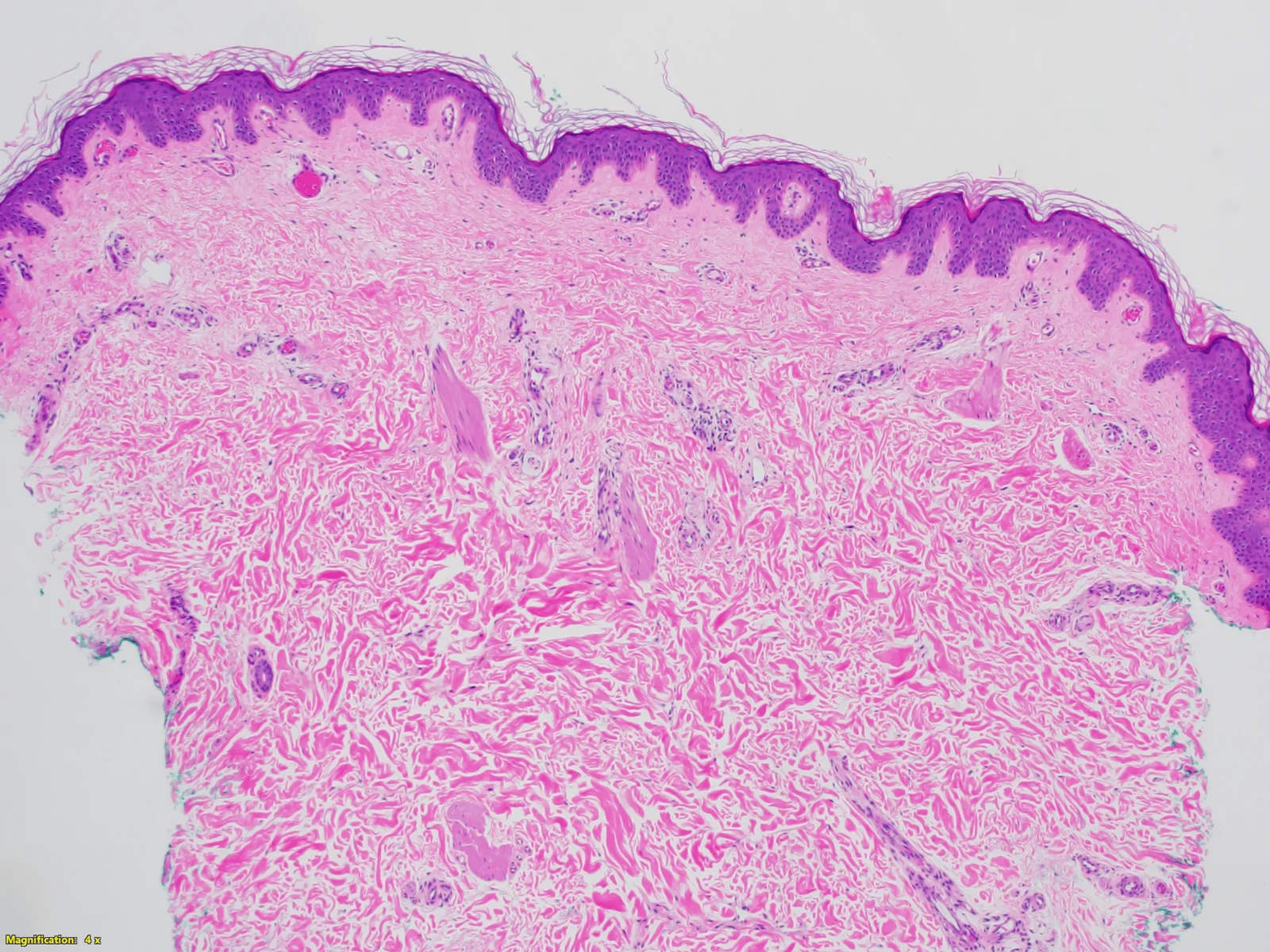
Histologic view showing a superficial vascular plexus with mildly dilated capillaries and sparse perivascular lymphocytic infiltrate (H&E, original magnification, 4×).

**Figure 4:**
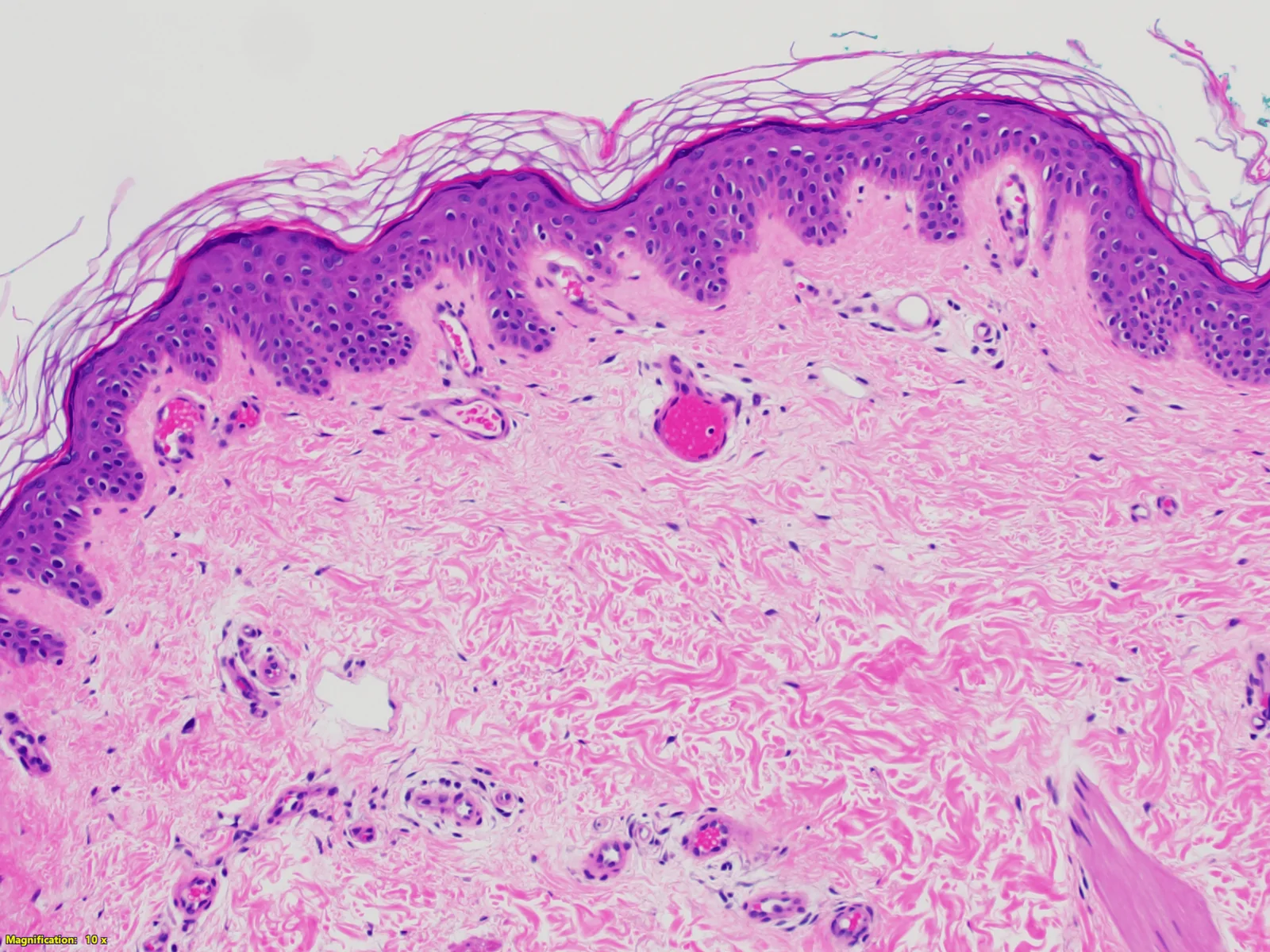
Histologic view showing a superficial vascular plexus with mildly dilated capillaries and sparse perivascular lymphocytic infiltrate (H&E, original magnification, 10×).

**Figure 5:**
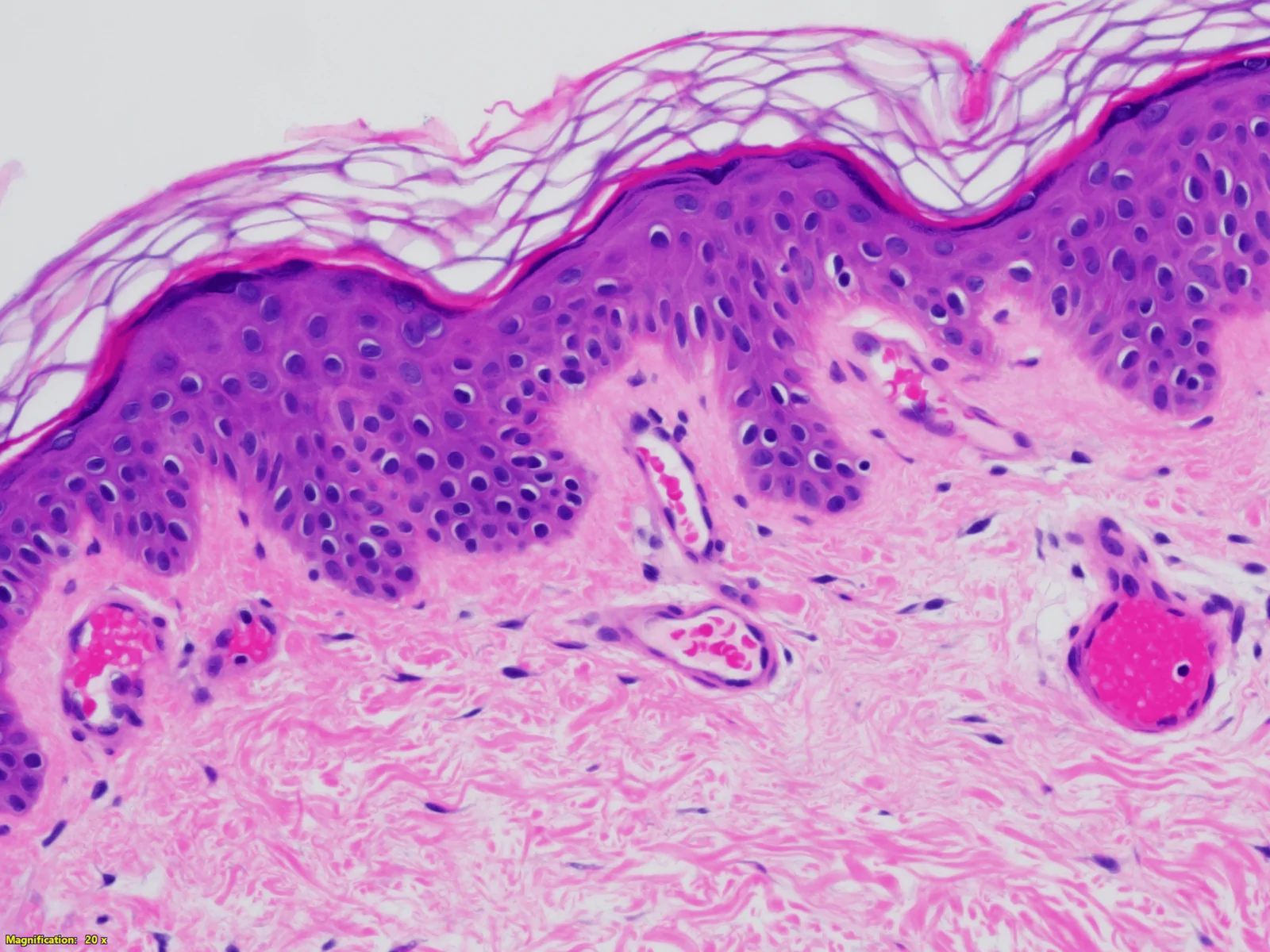
Histologic view showing a superficial vascular plexus with mildly dilated capillaries and sparse perivascular lymphocytic infiltrate (H&E, original magnification, 20×).

Two punch biopsy specimens were obtained from the left thigh. H&E sections showed mildly dilated superficial dermal capillaries with sparse lymphocytic inflammatory infiltrates, but no evidence of vascular occlusion, neutrophilia, leukocytoclasia, fibrin deposition, or vessel wall damage (Figure 3, Figure 4, Figure 5). A periodic acid-Schiff stain, performed to rule out fungal involvement, was negative. Direct immunofluorescence studies were negative for antibodies against IgG, IgA, IgM, fibrinogen, and C3. All relevant laboratory tests were normal (complete blood count, comprehensive metabolic panel, antinuclear antibody, antineutrophil cytoplasmic antibody, C-reactive protein, erythrocyte sedimentation rate, rheumatoid factor, lupus anticoagulant, anticardiolipin antibodies, and coagulation studies).

Direct pressure did not cause blanching, but injecting lidocaine with epinephrine into an involved area caused immediate blanching and temporary resolution of erythema. A diagnosis of AS was made and explained to the patient. She declined pulsed dye laser (PDL) therapy, which has been used to treat cosmetically distressing cases [4].

## Discussion

AS is a rare, benign vascular disorder characterized by multiple minute, nonblanching, red-purple macules arranged in a serpiginous or gyrate pattern [5]. It usually presents during childhood or adolescence and is more common among females. It typically presents as an acquired, unilateral eruption on the legs and buttocks. However, atypical presentations and extensive involvement have been reported in the literature [5,6].

Diagnosis of AS is primarily clinical, supported by dermoscopy that reveals well-demarcated red lagoons corresponding to dilated capillaries in the papillary dermis. Histopathology shows dilated, thin-walled capillaries without significant inflammation, red blood cell extravasation, or hemosiderin deposits [5,7,8]. AS can be differentiated from other purpuric dermatoses because it does not blanch under diascopy and lacks systemic involvement [5,7].

In general, treatment of AS is not necessary. Some patients, however, choose to treat their AS for cosmetic purposes [9]. PDL therapy is the treatment of choice, although potassium titanyl phosphate laser and intense pulsed light therapy have also shown efficacy in resolving AS lesions, with minimal post-treatment purpura or dyspigmentation [10].

The differential diagnosis for AS includes livedo racemosa, which was unlikely in this patient given the absence of thrombosis on histopathology. Livedo reticularis was also considered less likely, as it typically fluctuates with temperature, whereas these lesions were fixed. Leukocytoclastic vasculitis was unlikely due to the absence of vessel wall damage on histopathologic examination. Livedoid vasculopathy is the least common reported association with COVID-19 and appears mostly in elderly patients and those with severe COVID-19 infection [11], likely related to COVID-19-associated hypercoagulability. Recurrent livedoid vasculopathy triggered by COVID-19 infection was described in a 34-year-old woman without underlying comorbidities or coagulation abnormalities [12].

## Conclusions

Cutaneous manifestations of COVID-19 appear to occur more commonly in females and span a wide age range. Many of its cutaneous manifestations involve pathologic changes in the cutaneous vasculature, leading to relatively common associations (e.g., COVID toes) and more unusual or unexpected associations (e.g., retiform purpura). We describe a young woman who developed an uncommon presentation of AS during recovery from a COVID-19 infection. Her AS is also unusual because of its adult onset and bilateral symmetry. Given the growing recognition of vascular sequelae following COVID-19, our patient’s AS may represent another example of a COVID-19-associated acquired anomalous vasculopathy.
